# 1-Piperonylpiperazinium 4-chloro­benzoate

**DOI:** 10.1107/S1600536814002037

**Published:** 2014-02-12

**Authors:** Channappa N. Kavitha, Manpreet Kaur, Brian J. Anderson, Jerry P. Jasinski, H. S. Yathirajan

**Affiliations:** a Department of Studies in Chemistry, University of Mysore, Manasagangotri, Mysore 570 006, India; b Department of Chemistry, Keene State College, 229 Main Street, Keene, NH 03435-2001, USA

## Abstract

In the title salt {systematic name: 1-[(1,3-benzodioxol-5-yl)meth­yl]piperazin-1-ium 4-chloro­benzoate}, C_12_H_17_N_2_O_2_^+^·C_7_H_4_ClO_2_^−^, the piperazine ring adopts a slightly disordered chair conformation. The dioxole ring is in a flattened envelope conformation with the methyl­ene C atom forming the flap. The relative orientation of the piperonyl ring system and the piperazine rings is reflected in the N—C—C C torsion angle of 132.3 (1)°. In the anion, the mean plane of the carboxyl­ate group is twisted from that of the benzene ring by 14.8 (9)°. In the crystal, the components are linked by N—H⋯O and weak C—H⋯O hydrogen bonds, forming chains along [010].

## Related literature   

For the biological activity of related compounds, see: Brockunier *et al.* (2004 ▶); Bogatcheva *et al.* (2006 ▶); Elliott (2011 ▶); Gilbert *et al.* (1968 ▶); Gobert *et al.* (2003 ▶); Millan *et al.* (2001 ▶). For a related structure, see: Capuano *et al.* (2000 ▶). For puckering parameters, see: Cremer & Pople (1975 ▶). For standard bond lengths, see: Allen *et al.* (1987 ▶).
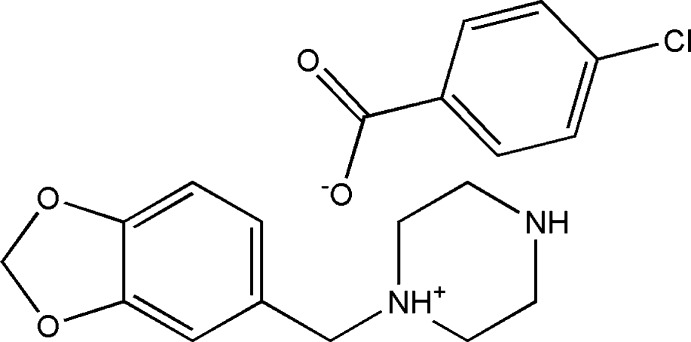


## Experimental   

### 

#### Crystal data  

C_12_H_17_N_2_O_2_^+^·C_7_H_4_ClO_2_^−^
*M* *_r_* = 376.83
Monoclinic, 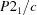

*a* = 16.9967 (6) Å
*b* = 8.5990 (3) Å
*c* = 12.4150 (5) Å
β = 90.923 (3)°
*V* = 1814.27 (12) Å^3^
*Z* = 4
Mo *K*α radiation
μ = 0.24 mm^−1^
*T* = 173 K
0.48 × 0.26 × 0.18 mm

#### Data collection  

Agilent Gemini EOS diffractometer
Absorption correction: multi-scan (*CrysAlis PRO* and *CrysAlis RED*; Agilent, 2012 ▶) *T*_min_ = 0.787, *T*_max_ = 1.000
22917 measured reflections
6302 independent reflections
4472 reflections with *I* > 2σ(*I*)
*R*_int_ = 0.033

#### Refinement  

*R*[*F* ^2^ > 2σ(*F* ^2^)] = 0.048
*wR*(*F* ^2^) = 0.120
*S* = 1.04
6302 reflections
235 parameters
H-atom parameters constrained
Δρ_max_ = 0.30 e Å^−3^
Δρ_min_ = −0.32 e Å^−3^

### 

Data collection: *CrysAlis PRO* (Agilent, 2012 ▶); cell refinement: *CrysAlis PRO*; data reduction: *CrysAlis RED* (Agilent, 2012 ▶); program(s) used to solve structure: *SUPERFLIP* (Palatinus & Chapuis, 2007 ▶); program(s) used to refine structure: *SHELXL2012* (Sheldrick, 2008 ▶); molecular graphics: *XP* in *SHELXTL* (Sheldrick, 2008 ▶) in *OLEX2* (Dolomanov *et al.*, 2009 ▶); software used to prepare material for publication: *OLEX2* (Dolomanov *et al.*, 2009 ▶).

## Supplementary Material

Crystal structure: contains datablock(s) I. DOI: 10.1107/S1600536814002037/lh5687sup1.cif

Structure factors: contains datablock(s) I. DOI: 10.1107/S1600536814002037/lh5687Isup2.hkl

Supporting information file. DOI: 10.1107/S1600536814002037/lh5687Isup3.cml

CCDC reference:

Additional supporting information:  crystallographic information; 3D view; checkCIF report

## Figures and Tables

**Table 1:** Hydrogen-bond geometry (Å, °)

*D*—H⋯*A*	*D*—H	H⋯*A*	*D*⋯*A*	*D*—H⋯*A*
N2*A*—H2*AA*⋯O1*B* ^i^	0.90	1.87	2.7606 (15)	171
N2*A*—H2*AB*⋯O2*B* ^ii^	0.90	1.78	2.6684 (16)	169
C10*A*—H10*A*⋯O2*B* ^iii^	0.97	2.57	3.1974 (17)	122

Symmetry codes: (i) 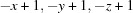; (ii) 
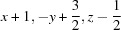
; (iii) 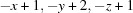.

**Fig. 1.**
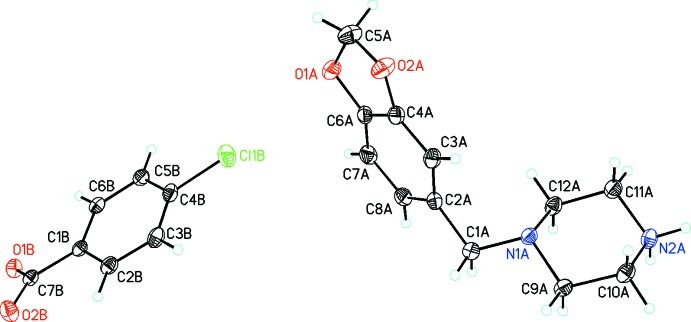
The asymmetric unit of (I) with 30% probability displacement ellipsoids.

**Fig. 2.**
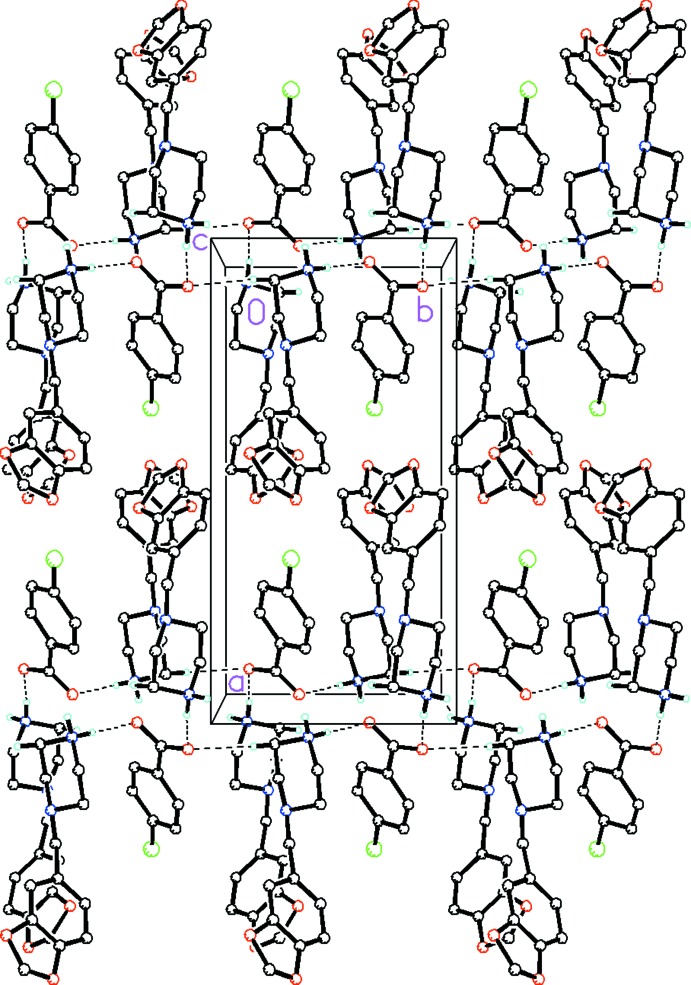
Crystal packing of (I) viewed along the c axis. Dashed lines indicate N—H···O and weak C—H···O interactions forming infinite 1-D chains along the b axis. H atoms not involved in hydrogen bonding have been removed for clarity.

## supplementary crystallographic information

### 1. Comment

1-(3,4-Methylenedioxybenzyl)piperazine or 1-piperonylpiperazine is a psychoactive drug of the piperazine class and is used to synthesise the drug, piribedil, an antiparkinsonian agent (Millan *et al.*, 2001). Piperonylpiperazine derivatives also have α-adrenergic antagonist properties (Gobert *et al.*, 2003) and peripheral vasodilator properties (Gilbert *et al.*, 1968). Piperazines are among the most important building blocks in today's drug discovery and are found in biologically active compounds across a number of different therapeutic areas (Brockunier *et al.*, 2004; Bogatcheva *et al.*, 2006). A review of the current pharmacological and toxicological information for piperazine derivatives is described (Elliott, 2011). The crystal structure of an N-piperonyl analogue of the atypical antipsychotic clozapine (Capuano *et al.*, 2000) is reported. In continuation of our work on salts of piperonylpiperazines, this paper reports the crystal structure of the title compound (I).

The asymmetric unit of (I) consists of a 1-piperonylpiperazinium cation and a p-chlorobenzoate anion (Fig. 1). The piperazine ring in the cation adopts a slightly disordered chair conformation (puckering parameters Q, θ, and φ = 0.5761 (14) Å , 177.7 (2) ° and 177 (4) °; (Cremer & Pople, 1975). The dioxole group is in a slightly distorted envelope configuration (puckering parameters Q and φ = 0.1693 (15) Å and 36.1 (5) ° with atom C5A displaced by 0.2683 (18) Å from the plane through the other four atoms). The piperonyl ring system and the piperazine rings are twisted with respect to each other as reflected in the N1A-C1A-C2A-C8A torsion angle of 132.2 (5)°. In the anion, the mean plane of the carboxylate group is twisted from that of the benzene ring by 14.8 (9)°. Bond lengths are in normal ranges (Allen *et al.*, 1987). In the crystal, N—H···O hydrogen bonds and a weak C10A—H10A···O2B^iii^ intermolecular interactions are observed which influence the crystal packing stability forming 1-D chains along [0 1 0] (Fig. 2).

### 2. Experimental

1-piperonylpiperazine ( 2.2 g, 0.01 mol) and p-chlorobenzoic acid (1.56 g, 0.01 mol) were dissolved in hot N,N-dimethylformamide and stirred for 10 mins at 323 K. The resulting solution was allowed to cool slowly at room temperature. The crystals of the title salt appeared after a few days and were suitable for X-ray studies (m.p.:464-470 K).

### 3. Refinement

All H atoms were placed in calculated positions and then refined using the riding-model approximation with Atom—H lengths of 0.93Å (CH), 0.97Å (CH_2_) or 0.90Å (NH). Isotropic displacement parameters were set to 1.2*U*_eq_ of the parent atom.

### Crystal data

**Table:**

C_12_H_17_N_2_O_2_^+^·C_7_H_4_ClO_2_^−^	*F*(000) = 792
*M**_r_* = 376.83	*D*_x_ = 1.380 Mg m^−^^3^
Monoclinic, *P*2_1_/*c*	Mo *K*α radiation, λ = 0.71073 Å
*a* = 16.9967 (6) Å	Cell parameters from 5056 reflections
*b* = 8.5990 (3) Å	θ = 3.1–32.8°
*c* = 12.4150 (5) Å	µ = 0.24 mm^−^^1^
β = 90.923 (3)°	*T* = 173 K
*V* = 1814.27 (12) Å^3^	Irregular, light yellow
*Z* = 4	0.48 × 0.26 × 0.18 mm

### Data collection

**Table:**

Agilent Gemini EOS diffractometer	6302 independent reflections
Radiation source: Enhance (Mo) X-ray Source	4472 reflections with *I* > 2σ(*I*)
Detector resolution: 16.0416 pixels mm^-1^	*R*_int_ = 0.033
ω scans	θ_max_ = 32.8°, θ_min_ = 3.1°
Absorption correction: multi-scan (*CrysAlis PRO* and *CrysAlis RED*; Agilent, 2012)	*h* = −21→25
*T*_min_ = 0.787, *T*_max_ = 1.000	*k* = −12→12
22917 measured reflections	*l* = −17→18

### Refinement

**Table:**

Refinement on *F*^2^	Primary atom site location: structure-invariant direct methods
Least-squares matrix: full	Hydrogen site location: mixed
*R*[*F*^2^ > 2σ(*F*^2^)] = 0.048	H-atom parameters constrained
*wR*(*F*^2^) = 0.120	*w* = 1/[σ^2^(*F*_o_^2^) + (0.0455*P*)^2^ + 0.5274*P*] where *P* = (*F*_o_^2^ + 2*F*_c_^2^)/3
*S* = 1.04	(Δ/σ)_max_ = 0.001
6302 reflections	Δρ_max_ = 0.30 e Å^−^^3^
235 parameters	Δρ_min_ = −0.32 e Å^−^^3^
0 restraints	

### Special details

**Table:**

Geometry. All esds (except the esd in the dihedral angle between two l.s. planes) are estimated using the full covariance matrix. The cell esds are taken into account individually in the estimation of esds in distances, angles and torsion angles; correlations between esds in cell parameters are only used when they are defined by crystal symmetry. An approximate (isotropic) treatment of cell esds is used for estimating esds involving l.s. planes.

### Fractional atomic coordinates and isotropic or equivalent isotropic displacement parameters (Å^2^)

**Table:**

	*x*	*y*	*z*	*U*_iso_*/*U*_eq_	
O1A	0.46343 (6)	0.65641 (14)	0.06488 (9)	0.0439 (3)	
O2A	0.55838 (7)	0.84302 (13)	0.03707 (9)	0.0452 (3)	
N1A	0.79257 (6)	0.70236 (13)	0.30762 (9)	0.0280 (2)	
N2A	0.95243 (6)	0.60800 (13)	0.27472 (10)	0.0298 (2)	
H2AA	0.9590	0.5293	0.3218	0.036*	
H2AB	0.9978	0.6209	0.2395	0.036*	
C1A	0.71612 (8)	0.69075 (19)	0.35969 (12)	0.0360 (3)	
H1AA	0.7072	0.7843	0.4014	0.043*	
H1AB	0.7170	0.6033	0.4090	0.043*	
C2A	0.64927 (8)	0.66996 (16)	0.27979 (11)	0.0301 (3)	
C3A	0.64318 (8)	0.77080 (16)	0.19132 (11)	0.0321 (3)	
H3A	0.6810	0.8466	0.1789	0.039*	
C4A	0.57955 (8)	0.75298 (16)	0.12438 (11)	0.0304 (3)	
C5A	0.49379 (11)	0.7623 (2)	−0.01222 (14)	0.0474 (4)	
H5AA	0.4533	0.8355	−0.0346	0.057*	
H5AB	0.5113	0.7063	−0.0753	0.057*	
C6A	0.52267 (8)	0.64112 (17)	0.14120 (11)	0.0312 (3)	
C7A	0.52767 (9)	0.54095 (18)	0.22546 (12)	0.0373 (3)	
H7A	0.4895	0.4654	0.2367	0.045*	
C8A	0.59275 (9)	0.55669 (18)	0.29434 (12)	0.0352 (3)	
H8A	0.5983	0.4885	0.3521	0.042*	
C9A	0.85456 (8)	0.73757 (16)	0.38737 (11)	0.0305 (3)	
H9AA	0.8570	0.6553	0.4408	0.037*	
H9AB	0.8422	0.8340	0.4239	0.037*	
C10A	0.93326 (8)	0.75244 (15)	0.33388 (12)	0.0313 (3)	
H10A	0.9320	0.8395	0.2842	0.038*	
H10B	0.9738	0.7727	0.3880	0.038*	
C11A	0.88861 (8)	0.56853 (18)	0.19658 (12)	0.0349 (3)	
H11A	0.9003	0.4701	0.1621	0.042*	
H11B	0.8854	0.6479	0.1412	0.042*	
C12A	0.81076 (8)	0.55708 (16)	0.25287 (12)	0.0316 (3)	
H12A	0.7695	0.5336	0.2005	0.038*	
H12B	0.8129	0.4730	0.3049	0.038*	
Cl1B	0.33436 (2)	0.68123 (5)	0.27279 (3)	0.04524 (12)	
O1B	0.02434 (6)	0.65334 (11)	0.59960 (8)	0.0335 (2)	
O2B	0.07906 (6)	0.87721 (13)	0.65085 (9)	0.0411 (3)	
C1B	0.14214 (7)	0.73384 (14)	0.51518 (10)	0.0255 (2)	
C2B	0.21013 (8)	0.82354 (16)	0.52310 (12)	0.0308 (3)	
H2B	0.2152	0.8966	0.5780	0.037*	
C3B	0.27034 (8)	0.80532 (16)	0.45013 (12)	0.0339 (3)	
H3B	0.3161	0.8642	0.4562	0.041*	
C4B	0.26105 (8)	0.69800 (16)	0.36822 (11)	0.0310 (3)	
C5B	0.19465 (8)	0.60586 (16)	0.35906 (11)	0.0311 (3)	
H5B	0.1897	0.5333	0.3038	0.037*	
C6B	0.13543 (8)	0.62363 (15)	0.43395 (11)	0.0284 (3)	
H6B	0.0909	0.5610	0.4296	0.034*	
C7B	0.07682 (8)	0.75601 (15)	0.59450 (11)	0.0275 (3)	

### Atomic displacement parameters (Å^2^)

**Table:**

	*U*^11^	*U*^22^	*U*^33^	*U*^12^	*U*^13^	*U*^23^
O1A	0.0290 (5)	0.0598 (7)	0.0426 (6)	−0.0056 (5)	−0.0065 (4)	0.0000 (5)
O2A	0.0446 (6)	0.0445 (6)	0.0461 (6)	−0.0058 (5)	−0.0140 (5)	0.0112 (5)
N1A	0.0247 (5)	0.0295 (5)	0.0297 (6)	0.0009 (4)	−0.0029 (4)	−0.0040 (4)
N2A	0.0248 (5)	0.0248 (5)	0.0398 (6)	0.0004 (4)	−0.0013 (4)	0.0035 (5)
C1A	0.0289 (7)	0.0482 (9)	0.0310 (7)	0.0017 (6)	0.0001 (5)	−0.0022 (6)
C2A	0.0243 (6)	0.0350 (7)	0.0311 (6)	0.0031 (5)	0.0028 (5)	−0.0031 (5)
C3A	0.0278 (6)	0.0295 (7)	0.0391 (7)	−0.0028 (5)	0.0005 (5)	−0.0004 (5)
C4A	0.0292 (6)	0.0292 (6)	0.0328 (7)	0.0029 (5)	0.0012 (5)	−0.0007 (5)
C5A	0.0497 (10)	0.0517 (10)	0.0403 (8)	−0.0057 (8)	−0.0118 (7)	−0.0001 (7)
C6A	0.0226 (6)	0.0374 (7)	0.0337 (7)	0.0002 (5)	0.0022 (5)	−0.0067 (6)
C7A	0.0312 (7)	0.0422 (8)	0.0387 (8)	−0.0097 (6)	0.0072 (6)	−0.0005 (6)
C8A	0.0341 (7)	0.0399 (8)	0.0317 (7)	−0.0015 (6)	0.0058 (5)	0.0035 (6)
C9A	0.0309 (7)	0.0293 (6)	0.0310 (6)	0.0010 (5)	−0.0055 (5)	−0.0025 (5)
C10A	0.0287 (6)	0.0239 (6)	0.0411 (7)	−0.0019 (5)	−0.0072 (5)	−0.0013 (5)
C11A	0.0304 (7)	0.0355 (7)	0.0385 (7)	0.0033 (5)	−0.0030 (6)	−0.0084 (6)
C12A	0.0276 (6)	0.0276 (6)	0.0393 (7)	−0.0002 (5)	−0.0049 (5)	−0.0056 (5)
Cl1B	0.0359 (2)	0.0490 (2)	0.0512 (2)	−0.00034 (16)	0.01216 (16)	0.00508 (18)
O1B	0.0296 (5)	0.0288 (5)	0.0421 (6)	−0.0036 (4)	0.0024 (4)	0.0014 (4)
O2B	0.0365 (6)	0.0362 (5)	0.0509 (6)	−0.0063 (4)	0.0073 (5)	−0.0127 (5)
C1B	0.0249 (6)	0.0224 (6)	0.0289 (6)	0.0012 (4)	−0.0045 (5)	0.0052 (5)
C2B	0.0282 (6)	0.0271 (6)	0.0369 (7)	−0.0019 (5)	−0.0059 (5)	−0.0003 (5)
C3B	0.0250 (6)	0.0303 (7)	0.0463 (8)	−0.0035 (5)	−0.0036 (5)	0.0042 (6)
C4B	0.0248 (6)	0.0321 (7)	0.0362 (7)	0.0033 (5)	0.0005 (5)	0.0084 (5)
C5B	0.0299 (6)	0.0313 (7)	0.0322 (7)	0.0020 (5)	−0.0043 (5)	−0.0003 (5)
C6B	0.0240 (6)	0.0281 (6)	0.0330 (7)	−0.0018 (5)	−0.0052 (5)	0.0030 (5)
C7B	0.0261 (6)	0.0253 (6)	0.0309 (6)	0.0024 (5)	−0.0046 (5)	0.0031 (5)

### Geometric parameters (Å, º)

**Table:**

O1A—C5A	1.424 (2)	C9A—H9AA	0.9700
O1A—C6A	1.3777 (17)	C9A—H9AB	0.9700
O2A—C4A	1.3753 (17)	C9A—C10A	1.508 (2)
O2A—C5A	1.4281 (19)	C10A—H10A	0.9700
N1A—C1A	1.4640 (17)	C10A—H10B	0.9700
N1A—C9A	1.4658 (16)	C11A—H11A	0.9700
N1A—C12A	1.4578 (17)	C11A—H11B	0.9700
N2A—H2AA	0.9000	C11A—C12A	1.510 (2)
N2A—H2AB	0.9000	C12A—H12A	0.9700
N2A—C10A	1.4818 (17)	C12A—H12B	0.9700
N2A—C11A	1.4829 (18)	Cl1B—C4B	1.7391 (14)
C1A—H1AA	0.9700	O1B—C7B	1.2573 (16)
C1A—H1AB	0.9700	O2B—C7B	1.2554 (16)
C1A—C2A	1.5069 (19)	C1B—C2B	1.3916 (18)
C2A—C3A	1.4019 (19)	C1B—C6B	1.3873 (18)
C2A—C8A	1.382 (2)	C1B—C7B	1.5079 (18)
C3A—H3A	0.9300	C2B—H2B	0.9300
C3A—C4A	1.3619 (19)	C2B—C3B	1.386 (2)
C4A—C6A	1.3820 (19)	C3B—H3B	0.9300
C5A—H5AA	0.9700	C3B—C4B	1.380 (2)
C5A—H5AB	0.9700	C4B—C5B	1.382 (2)
C6A—C7A	1.357 (2)	C5B—H5B	0.9300
C7A—H7A	0.9300	C5B—C6B	1.3896 (19)
C7A—C8A	1.394 (2)	C6B—H6B	0.9300
C8A—H8A	0.9300		
			
C6A—O1A—C5A	104.75 (11)	N1A—C9A—C10A	110.67 (11)
C4A—O2A—C5A	104.70 (12)	H9AA—C9A—H9AB	108.1
C1A—N1A—C9A	110.44 (11)	C10A—C9A—H9AA	109.5
C12A—N1A—C1A	110.11 (11)	C10A—C9A—H9AB	109.5
C12A—N1A—C9A	109.66 (10)	N2A—C10A—C9A	110.53 (11)
H2AA—N2A—H2AB	108.1	N2A—C10A—H10A	109.5
C10A—N2A—H2AA	109.5	N2A—C10A—H10B	109.5
C10A—N2A—H2AB	109.5	C9A—C10A—H10A	109.5
C10A—N2A—C11A	110.60 (10)	C9A—C10A—H10B	109.5
C11A—N2A—H2AA	109.5	H10A—C10A—H10B	108.1
C11A—N2A—H2AB	109.5	N2A—C11A—H11A	109.6
N1A—C1A—H1AA	109.1	N2A—C11A—H11B	109.6
N1A—C1A—H1AB	109.1	N2A—C11A—C12A	110.45 (12)
N1A—C1A—C2A	112.49 (11)	H11A—C11A—H11B	108.1
H1AA—C1A—H1AB	107.8	C12A—C11A—H11A	109.6
C2A—C1A—H1AA	109.1	C12A—C11A—H11B	109.6
C2A—C1A—H1AB	109.1	N1A—C12A—C11A	110.74 (11)
C3A—C2A—C1A	119.20 (13)	N1A—C12A—H12A	109.5
C8A—C2A—C1A	121.06 (13)	N1A—C12A—H12B	109.5
C8A—C2A—C3A	119.71 (13)	C11A—C12A—H12A	109.5
C2A—C3A—H3A	121.4	C11A—C12A—H12B	109.5
C4A—C3A—C2A	117.16 (13)	H12A—C12A—H12B	108.1
C4A—C3A—H3A	121.4	C2B—C1B—C7B	120.21 (12)
O2A—C4A—C6A	109.64 (12)	C6B—C1B—C2B	119.24 (12)
C3A—C4A—O2A	127.79 (13)	C6B—C1B—C7B	120.56 (11)
C3A—C4A—C6A	122.46 (13)	C1B—C2B—H2B	119.6
O1A—C5A—O2A	107.89 (12)	C3B—C2B—C1B	120.80 (13)
O1A—C5A—H5AA	110.1	C3B—C2B—H2B	119.6
O1A—C5A—H5AB	110.1	C2B—C3B—H3B	120.7
O2A—C5A—H5AA	110.1	C4B—C3B—C2B	118.69 (13)
O2A—C5A—H5AB	110.1	C4B—C3B—H3B	120.7
H5AA—C5A—H5AB	108.4	C3B—C4B—Cl1B	118.85 (11)
O1A—C6A—C4A	109.57 (13)	C3B—C4B—C5B	121.83 (13)
C7A—C6A—O1A	128.82 (13)	C5B—C4B—Cl1B	119.31 (11)
C7A—C6A—C4A	121.54 (13)	C4B—C5B—H5B	120.6
C6A—C7A—H7A	121.6	C4B—C5B—C6B	118.77 (13)
C6A—C7A—C8A	116.78 (13)	C6B—C5B—H5B	120.6
C8A—C7A—H7A	121.6	C1B—C6B—C5B	120.64 (12)
C2A—C8A—C7A	122.33 (14)	C1B—C6B—H6B	119.7
C2A—C8A—H8A	118.8	C5B—C6B—H6B	119.7
C7A—C8A—H8A	118.8	O1B—C7B—C1B	118.33 (12)
N1A—C9A—H9AA	109.5	O2B—C7B—O1B	124.75 (13)
N1A—C9A—H9AB	109.5	O2B—C7B—C1B	116.92 (12)
			
O1A—C6A—C7A—C8A	−176.43 (14)	C6A—C7A—C8A—C2A	1.1 (2)
O2A—C4A—C6A—O1A	0.07 (16)	C8A—C2A—C3A—C4A	1.3 (2)
O2A—C4A—C6A—C7A	−177.15 (13)	C9A—N1A—C1A—C2A	174.49 (12)
N1A—C1A—C2A—C3A	−49.87 (18)	C9A—N1A—C12A—C11A	−59.59 (14)
N1A—C1A—C2A—C8A	132.26 (14)	C10A—N2A—C11A—C12A	−55.32 (15)
N1A—C9A—C10A—N2A	−57.53 (14)	C11A—N2A—C10A—C9A	55.29 (14)
N2A—C11A—C12A—N1A	57.83 (15)	C12A—N1A—C1A—C2A	−64.26 (15)
C1A—N1A—C9A—C10A	−179.02 (11)	C12A—N1A—C9A—C10A	59.46 (14)
C1A—N1A—C12A—C11A	178.69 (11)	Cl1B—C4B—C5B—C6B	−178.09 (10)
C1A—C2A—C3A—C4A	−176.59 (13)	C1B—C2B—C3B—C4B	1.0 (2)
C1A—C2A—C8A—C7A	175.95 (13)	C2B—C1B—C6B—C5B	−2.03 (19)
C2A—C3A—C4A—O2A	175.68 (13)	C2B—C1B—C7B—O1B	−165.32 (12)
C2A—C3A—C4A—C6A	0.0 (2)	C2B—C1B—C7B—O2B	14.80 (18)
C3A—C2A—C8A—C7A	−1.9 (2)	C2B—C3B—C4B—Cl1B	177.00 (10)
C3A—C4A—C6A—O1A	176.46 (13)	C2B—C3B—C4B—C5B	−1.9 (2)
C3A—C4A—C6A—C7A	−0.8 (2)	C3B—C4B—C5B—C6B	0.8 (2)
C4A—O2A—C5A—O1A	18.16 (18)	C4B—C5B—C6B—C1B	1.22 (19)
C4A—C6A—C7A—C8A	0.2 (2)	C6B—C1B—C2B—C3B	0.89 (19)
C5A—O1A—C6A—C4A	11.18 (16)	C6B—C1B—C7B—O1B	14.36 (18)
C5A—O1A—C6A—C7A	−171.86 (16)	C6B—C1B—C7B—O2B	−165.52 (12)
C5A—O2A—C4A—C3A	172.60 (15)	C7B—C1B—C2B—C3B	−179.42 (12)
C5A—O2A—C4A—C6A	−11.25 (16)	C7B—C1B—C6B—C5B	178.29 (11)
C6A—O1A—C5A—O2A	−18.11 (17)		

### Hydrogen-bond geometry (Å, º)

**Table:**

*D*—H···*A*	*D*—H	H···*A*	*D*···*A*	*D*—H···*A*
N2*A*—H2*AA*···O1*B*^i^	0.90	1.87	2.7606 (15)	171
N2*A*—H2*AB*···O2*B*^ii^	0.90	1.78	2.6684 (16)	169
C10*A*—H10*A*···O2*B*^iii^	0.97	2.57	3.1974 (17)	122

Symmetry codes: (i) −*x*+1, −*y*+1, −*z*+1; (ii) *x*+1, −*y*+3/2, *z*−1/2; (iii) −*x*+1, −*y*+2, −*z*+1.
